# Nasogastric tube combined with thin therapeutic endoscope to facilitate esophageal endoscopic submucosal dissection

**DOI:** 10.1055/a-2421-9676

**Published:** 2024-10-15

**Authors:** Yuka Kowazaki, Hisashi Fukuda, Tetsurou Miwata, Takaaki Morikawa, Sawako Fujikura, Jun Ushio

**Affiliations:** 1 Department of Gastroenterology, Jyoban Hospital, Tokiwa Foundation, Iwaki, Japan; 2 12838 Department of Medicine, Division of Gastroenterology, Jichi Medical University, Shimotsuke, Japan; 3 Department of Gastroenterology and Hepatology, Mie University Hospital, Tsu, Japan; 4 378609 Department of Digestive Disease Center, Showa University Koto Toyosu Hospital, Koto-ku, Japan

Esophageal endoscopic submucosal dissection (ESD) has recently been widely performed to treat superficial esophageal cancer without lymph node metastasis or with a low risk of metastasis 1 . During esophageal ESD, air accumulates in the stomach ( Fig. 1 ), which can cause a vagovagal reflex, resulting in vital sign changes such as bradycardia and hypotension. Even under sedation, patients complain of distress owing to the presence of air in the stomach, which results in increased body movement and sedative dosing. Moreover, a dilated stomach may lead to the complication of Mallory-Weiss syndrome during ESD 2 .Thus, gastric air and fluid must be aspirated several times while performing esophageal ESD. The situation is similar in cases of gastric and colorectal ESD; frequent aspiration is time consuming and challenging.

**Fig. 1:**
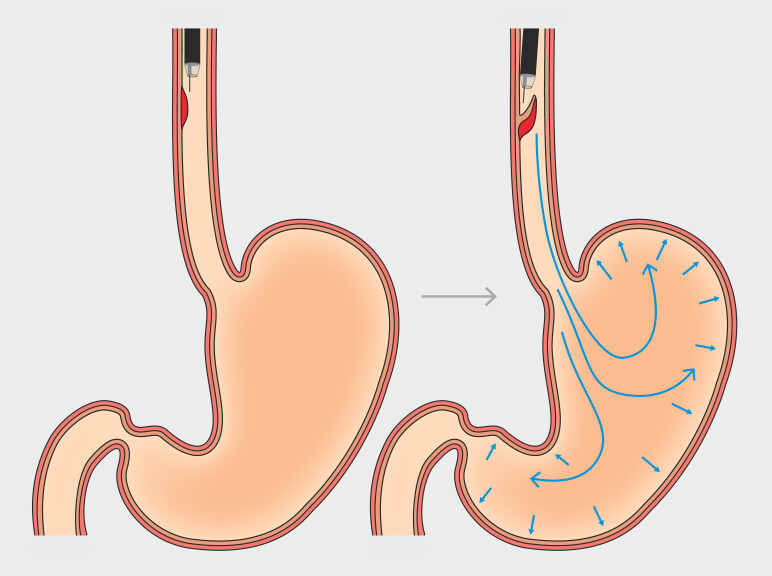
During conventional esophageal ESD, air accumulates in the stomach.

Therefore, we developed a method for gastric ESD involving the use of a nasogastric tube 3 . Tube placement during ESD has been reported to be helpful for treating large rectal tumors 4 . Hence, we considered using a nasogastric tube for esophageal ESD as a more efficient treatment option ( Fig. 2 ).

**Fig. 2:**
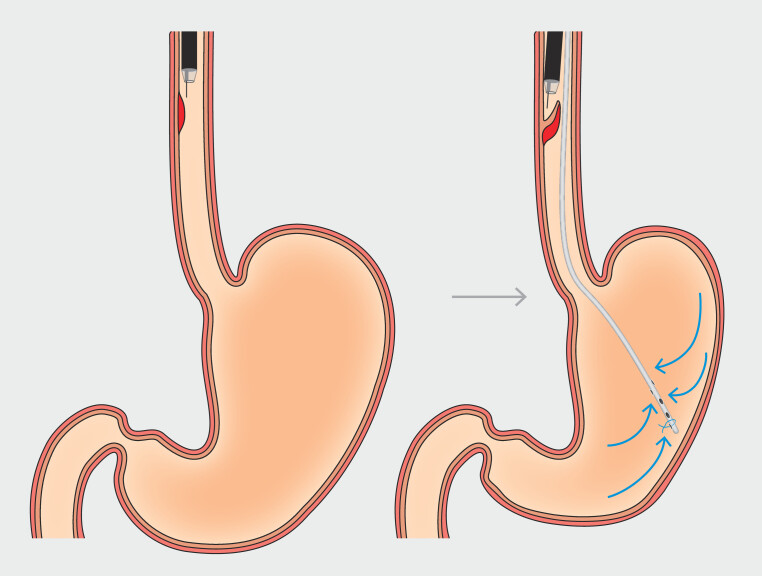
When esophageal ESD is performed using a nasogastric tube, the stomach remains collapsed.

A 14F nasogastric tube (TOP Co., Tokyo, Japan) with a 3–0 nylon loop at the tip ( Fig. 3 ) was inserted through the nasal cavity and clipped to the greater curvature of the gastric body ( Fig. 4 ). To minimize interference between the endoscope and the nasogastric tube, esophageal ESD was performed using a thin therapeutic endoscope (EG-840TP, Fujifilm Co., Tokyo, Japan), with an outer diameter of only 7.9 mm but an accessory channel diameter of 3.2 mm ( Fig. 5 ) 5 . Air and fluid naturally drained from the stomach through the nasogastric tube; therefore, scope insertion into the stomach to aspirate air during ESD was not needed. Esophageal ESD was performed without any complications ( Video 1 ).

**Fig. 3:**
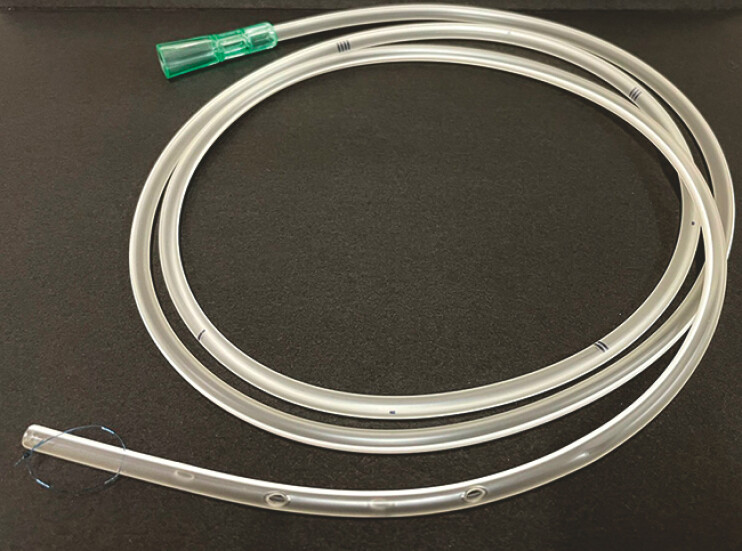
A 14F nasogastric tube (TOP Co., Tokyo, Japan) with a 3–0 nylon loop at the tip.

**Fig. 4:**
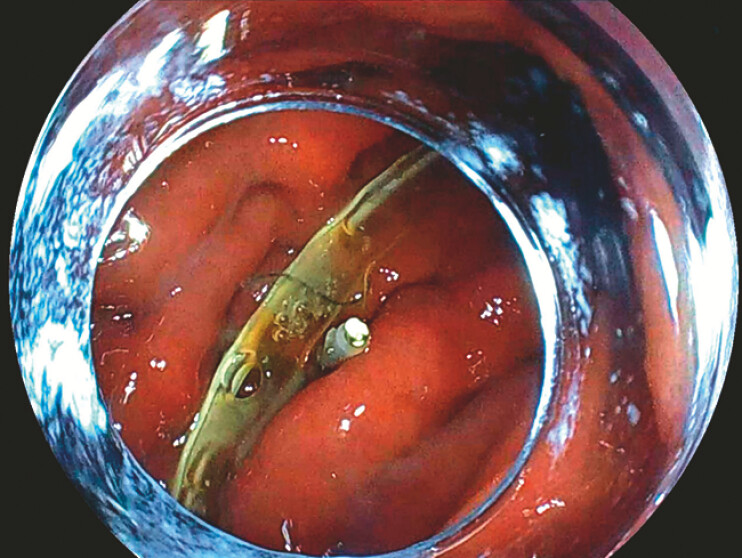
Endoscopic image of the nasogastric tube clipped to the greater curvature of the gastric body.

**Fig. 5:**
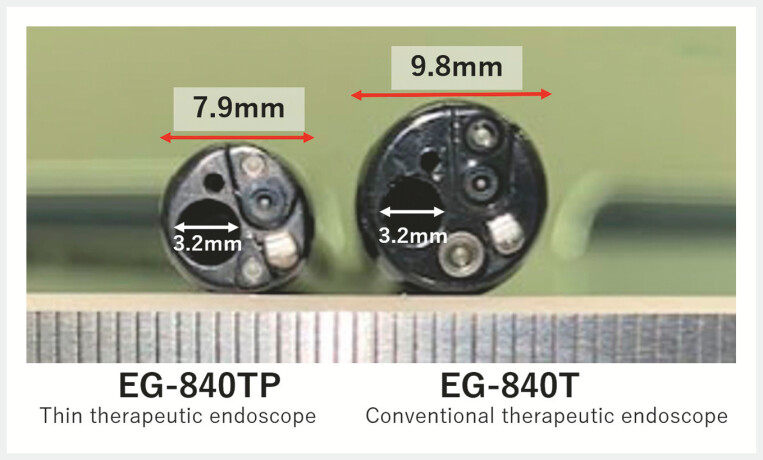
A thin therapeutic endoscope (EG-840TP, Fujifilm Co., Tokyo, Japan) compared with a conventional therapeutic endoscope.

A nasogastric tube combined with a thin therapeutic endoscope to facilitate esophageal endoscopic submucosal dissection.Video 1

## Conclusions

In conclusion, esophageal ESD using a nasogastric tube is safe and convenient.
